# Effects of Melatonin and Its Analogues on Pancreatic Inflammation, Enzyme Secretion, and Tumorigenesis

**DOI:** 10.3390/ijms18051014

**Published:** 2017-05-08

**Authors:** Jolanta Jaworek, Anna Leja-Szpak, Katarzyna Nawrot-Porąbka, Joanna Szklarczyk, Michalina Kot, Piotr Pierzchalski, Marta Góralska, Piotr Ceranowicz, Zygmunt Warzecha, Artur Dembinski, Joanna Bonior

**Affiliations:** 1 Department of Medical Physiology, Faculty of Health Sciences, Jagiellonian University Medical College, 31-126 Kraków, Poland; jolanta.jaworek@uj.edu.pl (J.J.); a.leja-szpak@uj.edu.pl (A.L.-S.); k.nawrot-porabka@uj.edu.pl (K.N.-P.); joannam.szklarczyk@uj.edu.pl (J.S.); michalina.kot@uj.edu.pl (M.K.); piotr.pierzchalski@uj.edu.pl (P.P.); marta.goralska@uj.edu.pl (M.G.); joanna.bonior@uj.edu.pl (J.B.); 2 Department of Physiology, Faculty of Medicine, Jagiellonian University Medical College, 31-531 Kraków, Poland; zygmunt.warzecha@uj.edu.pl (Z.W.); artur.dembinski@uj.edu.pl (A.D.)

**Keywords:** melatonin, AFMK, enzyme secretion, acute pancreatitis, pancreatic cancer

## Abstract

Melatonin is an indoleamine produced from the amino acid l-tryptophan, whereas metabolites of melatonin are known as kynuramines. One of the best-known kynuramines is *N*^1^-acetyl-*N*^1^-formyl-5-methoxykynuramine (AFMK). Melatonin has attracted scientific attention as a potent antioxidant and protector of tissue against oxidative stress. l-Tryptophan and kynuramines share common beneficial features with melatonin. Melatonin was originally discovered as a pineal product, has been detected in the gastrointestinal tract, and its receptors have been identified in the pancreas. The role of melatonin in the pancreatic gland is not explained, however several arguments support the opinion that melatonin is probably implicated in the physiology and pathophysiology of the pancreas. (1) Melatonin stimulates pancreatic enzyme secretion through the activation of entero-pancreatic reflex and cholecystokinin (CCK) release. l-Tryptophan and AFMK are less effective than melatonin in the stimulation of pancreatic exocrine function; (2) Melatonin is a successful pancreatic protector, which prevents the pancreas from developing of acute pancreatitis and reduces pancreatic damage. This effect is related to its direct and indirect antioxidant action, to the strengthening of immune defense, and to the modulation of apoptosis. Like melatonin, its precursor and AFMK are able to mimic its protective effect, and it is commonly accepted that all these substances create an antioxidant cascade to intensify the pancreatic protection and acinar cells viability; (3) In pancreatic cancer cells, melatonin and AFMK activated a signal transduction pathway for apoptosis and stimulated heat shock proteins. The role of melatonin and AFMK in pancreatic tumorigenesis remains to be elucidated.

## 1. Melatonin, Its Precursor and Derivatives 

Melatonin (*N*-acetyl-5-methoxytryptamine) is the main product of the pineal gland, and remains an intriguing substance. Its physiological role is still unclear. This indoleamine is widely distributed among living organisms and has been detected in all living species, from bacteria and plants to mammals [1,2].

Melatonin is able to influence several functions of the organism, including regulation of circadian rhythms, modulation of immune response and hormones release, and reduction of the radical burden of tissues [3,4,5,6]. This substance is known as an extremely potent protector of tissues that effectively combats oxidative stress and saves the organism from damage induced by toxic radicals [6,7]. Tissue protection afforded by melatonin is related to its double antioxidant actions, including: (1) Direct neutralization of devastative reactive oxygen and nitrogen species (ROS and RNS); and (2) Indirect effect through the activation of antioxidant enzymes—superoxide dismutase (SOD), catalase (CAT), glutathione peroxidase (GPx), or glutathione reductase (GR) [7,8,9,10]. In addition, melatonin modulates the inflammatory defense of the organism, decreasing the production of pro-inflammatory cytokines such as interleukin 1β (IL-1β), interleukin 6 (IL-6), interleukin 22 (IL-22), tumor necrosis factor α (TNFα) and increasing the anti-inflammatory interleukin 10 (IL-10) [11,12,13,14,15,16]. Melatonin is able to control the above-mentioned processes at the cellular level via inhibition of NF-κB, a nuclear transcription factor responsible for the control of genes involved in cell apoptosis and the production of inflammatory mediators, whereas the activation of antioxidant enzymes by melatonin is possibly related to the stimulation of protein kinase C (PKC) and Ca^2+^ influx via the induction and nuclear translocation of nuclear transcription factor Nrf1 [12,13,17].

The biological effects of melatonin are mediated by specific membrane receptors MT_1_, MT_2_/MT_3_ and probably by its nuclear orphan receptors: retinoid-related orphan receptors and retinoid Z receptors (ROR/RZR) [18,19,20]. Transmembrane G-protein-coupled receptors MT_1_ and MT_2_ share 60% homology and could be antagonized by luzindole [21]. Melatonin receptor MT_3_ has been identified as an enzyme quinone reductase 2 (QR2) and it may be partially responsible for the antioxidant effects of melatonin, whereas the role of nuclear orphan receptors ROR/RZR is unknown [18,19]. Besides its receptor-mediated action, melatonin is capable of free penetration of the cellular membrane barrier, because it is highly lipophilic and this property enables melatonin to effectively protect the cell compartment against oxidative damage [22,23].

Melatonin is produced from the amino acid l-tryptophan, which is converted to serotonin and subsequently to melatonin in processes involving the enzymes arylalkylamino-*N*-acetyl-serotonin-transferase (AA-NAT) and hydroxyindolo-*O*-methyl-transferase (HIOMT) [18,24,25]. The gene expression of both enzymes has been identified in the gut and in the pancreas [18,24,25,26,27]. l-tryptophan has been shown to protect the tissues in a way similar to its derivative, melatonin [26].

Melatonin can be metabolized in several ways, involving deacetylation, cytochrome P450s-mediated processes, and classic kynurenic pathway, with key enzyme indoleamine 2,3-dioxygenase (IDO) [28,29]. The metabolic products of melatonin and of l-tryptophan are known as kynuramines. Among them, the best-known melatonin derivatives are *N*^1^-acetyl-*N*^1^-formyl-5-methoxykynuramine (AFMK), and *N*^1^-acetyl-5-methoxy-kynuramine (AMK). These molecules can be generated from melatonin by myelo- or hemoperoxidase enzymes, and also by free radicals or by ultraviolet radiation [30]. Like its maternal molecule, AFMK has been demonstrated as an effective cell protector against oxidative stress. It is able to neutralize reactive molecules (mainly hydroxyl (•OH) radical) and can reduce membrane lipid peroxidation and DNA damage; however, its scavenging effect is weaker than the antioxidant properties of melatonin [31,32,33,34,35]. AFMK has been found in many organisms (including mammals, where it was identified in the eye, in the brain, and in the skin); however, this melatonin derivative was not detected in the serum under normal conditions [30,31,32]. It has been suggested that an increased formation of AFMK from melatonin could be indicative of activation of inflammatory processes, and indeed AFMK was identified in the cerebrospinal fluid of patients with meningitis [29,36]. AFMK was markedly increased in the human epidermal keratinocytes exposed to UVB radiation, which is consistent with the photoprotective role of melatonin and its metabolites [32].

## 2. Melatonin and Its Receptors in the Pancreas

Melatonin—previously identified as an exceptional pineal product—can be synthetized in many other tissues, such as retina, brain, heart, blood vessels, immune cells, and the gastrointestinal system [18,34,37,38,39,40]. The main source of melatonin is probably the gastrointestinal tract, with the estimated amount of produced melatonin ranging 400 times higher than that in the pineal gland [37,39,40].

It is of interest that the pancreas is probably also able to synthetize melatonin, as was evidenced by the presence of mRNA signals for AA-NAT and HIOMT in the rat pancreatic acini and in human pancreatic tissue [26,27,40]. Melatonin concentration in the pancreatic tissue undergoes circadian fluctuation and mimics the characteristic changes of melatonin in the pineal gland, with a higher amount during the dark phase and lower during the day (10 and 5 pg/100 mg of tissue, respectively) [20]. These changes are independent of the pineal gland, but can be associated with ingested food [41].

Melatonin receptors have been identified in the pancreatic islets of Langerhans (in β-cells and in α-cells), in the azaserine-induced malignant nodules from the rat pancreas (pancreatic acinar cell line) AR42J, and in the human pancreatic cancer cell line PANC-1 [42,43,44].

## 3. Role of Melatonin and Its Analogues in Pancreatic Enzyme Secretion

Previous experimental studies have demonstrated that melatonin and its precursor l-tryptophan given parenterally or into the duodenal lumen produced significant increases of pancreatic enzymes secretion [27,45,46]. Since both key enzymes involved in the transformation of l-tryptophan into melatonin were detected in the cells of gut mucosa and the application of l-tryptophan resulted in a dose-dependent rise of melatonin blood level, it could be presumed that l-tryptophan itself is not responsible for the stimulation of the pancreatic enzymes, but melatonin derived from its precursor in the gut lumen.

Melatonin appears as a very potent pancreatic secretagogue. A 25 mg/kg dose of melatonin given intraduodenally produced enzyme secretion similar to the level achieved by the administration of 1 µg/kg of cholecystokinin (CCK) (near maximal secretion) [45]. The pancreatostimulatory effect of melatonin was much stronger with intraluminal administration of this ingredient when compared to the increased pancreatic enzyme secretion evoked by its application into the blood stream [45]. This observation indicates that the stimulatory effect of melatonin and related substances is dependent on the activation of receptors located in the intestine rather than in the pancreatic gland. This suggestion was supported by the observation that melatonin failed to produce the secretory response from isolated rat pancreatic acini. A controversial observation was published by Santofimia-Castaño, P. et al. [47], who demonstrated that melatonin reduced calcium mobilization and attenuated amylase release stimulated by CCK in mouse pancreatic acini. The differences between our results and the observation of Santofimia-Castaño, P. may be explained by variances between species used in the in vitro experiments, or perhaps by a competitive inhibitory effect of melatonin and CCK on calcium mobilization and enzyme secretion.

The mechanism of the stimulatory action of melatonin on pancreatic exocrine function appears indirect and in need of the release of CCK, because application of melatonin or its precursor resulted in dose-dependent increase of CCK blood level. In addition, melatonin-evoked pancreatic secretory response was completely blocked by CCK receptor antagonists such as tarazepide or L-364,718, as well as by deactivation of sensory nerves by capsaicin. This observation leads to the conclusion that stimulation of pancreatic enzyme secretion evoked by intraluminal melatonin is dependent on the release of CCK and activation (directly or indirectly, via CCK) of the duodeno-pancreatic reflex [46]. 

Melatonin is present in the gut lumen as a component of ingested food, or as derivative of l-tryptophan and serotonin [48,49]. It is very likely that melatonin could play an essential role in the physiological stimulation of exocrine pancreatic function. Melatonin possibly acts as one of the mediators involved in the intestinal phase of pancreatic secretion via the activation of the neural entero-pancreatic reflex. l-tryptophan seems to function as a melatonin precursor, whereas role of AFMK in the regulation of pancreatic exocrine function remains to be clarified.

## 4. Melatonin System and Acute Pancreatitis 

Acute pancreatitis is a primary sterile disease of the pancreas, classified as edematous or hemorrhagic-necrotizing form, according to the morphological changes of the pancreatic tissue. Edematous pancreatitis has a good prognosis, but the severe hemorrhagic-necrotizing type of pancreatic inflammation is characterized by high mortality rate [50,51]. The pathogenesis of acute pancreatitis is not completely explained, and the main mechanism has been based on the concept of pancreas self-digestion, being the consequence of the intracellular activation of pancreatic digestive enzymes (mainly trypsin) leading to acinar cell necrosis [52]. Toxic ingredients released from the damaged structures such as ROS and RNS, products of arachidonic acid metabolism (prostaglandins, leukotrienes, thromboxanes), and other noxious agents work as propagators of sterile inflammation and chemoattractants for inflammatory cells, leading to the increased production of pro-inflammatory cytokines (e.g., TNFα, IL-6, IL-8). This is accompanied by vascular endothelium damage, increased vascular permeability, and processes of intensified coagulation and fibrinolysis [52,53,54,55]. Local pancreatic inflammation could develop into systemic inflammatory response (SIRS), often leading to multiple organ dysfunction (MODS) and septic shock [55,56].

The activation of proinflammatory mechanisms in acute pancreatitis is followed by the mobilization of the compensatory anti-inflammatory response syndrome of the organism (CARS), which could limit or suppress the process of inflammation. The prevalence of pro-inflammatory agents leads to the propagation of acute pancreatitis, whereas stimulation of natural defense mechanisms results in the suppression of inflammatory process [57,58].

Melatonin has been recognized as one of the elements of the innate defense system and a very effective pancreatic protector [2,59]. Previous experimental studies have shown that pretreatment with melatonin could prevent pancreatic inflammation and radically reduce pancreatic tissue damage in rats subjected to acute pancreatitis in different experimental models [13,59,60,61,62,63,64,65,66,67,68,69,70,71,72,73]. This was evidenced by histological assessment of pancreatic tissue and prominent decline of the inflammatory markers, such as edema, neutrophil infiltration, and vacuolization of the acinar cells [60,61,62,63,73]. In animals with acute pancreatitis pretreated with melatonin, there was a significant reduction in the plasma morphometric indicators of pancreatic inflammation severity, blood levels of the enzymes, amylase and lipase, and blood levels of pro-inflammatory cytokines (e.g., TNFα, IL-1β, and IL-6), whereas anti-inflammatory interleukins IL-10 and IL-4 were increased [10,59,62,63,64,65,66,67,68,69,70]. The antioxidant effect of melatonin was expressed as a reduction of lipid peroxidation products (MDA + 4-HNE) in the pancreas and was accompanied by increased activity of antioxidant enzymes SOD, CAT, GPx, and GSH [62,63,64,65,66,67,69,70]. Other mechanisms of the protective action of melatonin on acute pancreatitis involve the improvement of pancreatic blood flow, the reduction of prostaglandin generation, a decrease in myeloperoxidase (MPO), the moderation of apoptosis and necrosis processes in the pancreatic tissue, and synthesis of heat shock proteins [63,66,69,72,74]. Moreover, melatonin has been shown to protect against lung injury and to save gut barrier integrity to reduce bacterial translocation and to prevent sepsis and MODS in acute pancreatitis [75,76]. Furthermore, the recovery from acute pancreatitis and pancreatic regeneration was improved by melatonin [77].

This potent favorable effect of melatonin on acute pancreatitis is not related to an individual action of melatonin, but perhaps depends on the involvement of a whole melatonin system with the contribution of the melatonin precursor l-tryptophan as well as the participation of melatonin derivatives. Our recent study has shown that one of them—melatonin metabolite AFMK—is also able to attenuate the severity of acute pancreatic inflammation. Administration of AFMK to rats prior to the induction of caerulein-induced pancreatitis significantly reduced pancreatic tissue damage in a way similar to its maternal molecule, melatonin. AFMK reduced the morphometric signs of inflammation, decreased amylase serum activity and TNFα serum concentration, and increased the antioxidant potency of pancreatic tissue, which was evidenced by the rise of the antioxidant enzyme GPx in the pancreas, accompanied by the significant decline of pancreatic lipid peroxidation products MDA + 4-HNE [78].

Looking for the protective mechanisms of melatonin and AFMK at the molecular level, we have observed that both substances stimulated the protein expression of GPx, while reducing that of TNFα in the acinar pancreatic cell line AR42J subjected to high doses of caerulein. In addition, AFMK and its maternal molecule modulated the apoptotic signal transduction pathway in these cells, leading to the activation of the proapoptotic enzyme caspase-3 [78]. Apoptosis (programmed cell death) prevents cell membranes from interruption and appears as a process limiting the inflammatory reactions in the tissues; this is unlike necrosis, which destroys tissue by rupturing cellular membranes and releases lysosomal enzymes and inflammatory mediators outside of the cell, facilitating the propagation of inflammation [79,80]. Melatonin has previously been demonstrated to improve signals for proapoptotic enzymes caspase-3 and caspase-7 in cancer cells [81,82,83]. In the pancreatic cell line, both melatonin and AFMK enhanced proapoptotic Bax protein and reduced signals for antiapoptotic Bcl-2, and this was followed by the rise of active caspase-3, executor of apoptosis [82].

Another protective mechanism of melatonin and AFMK in the pancreas could be related to the suppression of inducible nitric oxide synthase (iNOS) activity. This isoenzyme is responsible for excessive generation of nitric oxide (NO) and the formation of toxic RNS in acute pancreatitis [13]. Melatonin has been demonstrated to diminish the expression of iNOS in the pancreas of rats subjected to acute pancreatitis. Like its precursor molecule, AFMK also prevented activation of iNOS with subsequent reduction of NO concentration in activated macrophages [34]. This effect could possibly be involved in the alleviation of inflammatory processes afforded by the melatonin system in the pancreas.

Results of experimental studies presented evidence that melatonin may take part in the natural defense system improving pancreatic resistance against inflammation, and that the elimination of endogenous melatonin by the application of an antagonist of melatonin MT_1_/MT_2_ receptors (luzindole) worsened the course of acute pancreatitis and increased lipid peroxidation in the inflamed pancreas [63]. Another argument supporting the role of endogenous melatonin in the innate pancreatic defense came from the observation that in pinealectomized rats subjected to acute pancreatitis, the inflammatory changes of the gland were significantly aggravated compared to animals with intact pineal gland subjected to pancreatic inflammation [84].

The possible implication of melatonin in the innate defense of the pancreas against acute inflammation was supported by clinical observations, showing that in patients with acute pancreatitis, high melatonin blood concentration observed in the first 24 h after the onset of pancreatic inflammation was correlated with a mild course and optimistic prognosis for patients, whereas severe pancreatitis was accompanied with low melatonin blood level [85,86].

All above results of experimental studies together with clinical observations supported the notion of the important role of the melatonin system in the innate defense protecting the pancreatic gland from inflammatory damage. The prolonged and effective pancreatic protection results from the common beneficial action of melatonin, its precursor, and its metabolites. All these substances create an antioxidant cascade, and work together to increase antioxidant resistance of pancreatic tissue and to protect acinar cells viability.

## 5. Melatonin System and Pancreatic Cancer

The results of our previous study provided the evidence that melatonin given to the pancreatic cancer cells PANC-1 activated the pro-apoptotic signal transduction pathway and stimulated the expression of caspase-9, thus activating the mitochondrial pathway of apoptosis. However, the activation of executor of apoptosis (caspase-3) and DNA fragmentation have not been observed, indicating that the apoptotic process might not be completed [43,87,88]. The pro-apoptotic effect of melatonin on pancreatic tumor cells has been demonstrated by Li et al. and Xu et al., who have shown that the application of melatonin to cancer cells resulted in the strong expression of pro-apoptotic Bax proteins followed by the activation of caspase-3 and inhibition of an anti-apoptotic protein Bcl-2 [82,89]. Similar results have been demonstrated in the pancreatic AR42J tumor acinar cells. Melatonin reduced the viability of these cells via the activation of caspase-3 and modulation of mitochondrial activity [44]. Moreover, melatonin has been demonstrated to up-regulate Bax/Bcl-2 or Bax/Bcl-xL proteins ratio followed by activation of caspase-9 and caspase-7 in the cultures of breast, lung, and colon cancer cells [90,91,92].

The effects of melatonin on pancreatic cancer cells could be related to the activation of melatonin membrane receptors (MT_1_/MT_2_), because pretreatment with luzindole (antagonist of these receptors) reversed the stimulatory effect of melatonin on Bcl-2/Bax balance and caspase-9 proteins expression in PANC-1 cells [43].

To the best of our knowledge, only a few observations concerning the effects of melatonin metabolite AFMK on pancreatic tumor cells have been published to date. A single study of Kim et al. demonstrated that melatonin derivatives such as 6-hydroxymelatonin, AFMK, and 5-methoxytryptamine inhibited the proliferation of human normal epidermal melanocytes and melanoma cells [93]. Our recent publication showed that AFMK given to human pancreatic carcinoma cells (PANC-1) increased the signal for the anti-apoptotic heat shock proteins nuclear p-HSP27, cytoplasmic HSP90α/β, and HSP70. These effects could be related to the activation of melatonin membrane receptors by AFMK, because luzindole (antagonist of these receptors) reversed AFMK-induced stimulation of HSPs in PANC-1 cells [94]. The observation concerning the involvement of MT_1_/MT_2_ receptor in the effect of AFMK is in agreement with previous reports, showing that AFMK is able to bind to the melatonin membrane receptor, but its affinity to this receptor is lower than that of melatonin [34,95,96,97,98,99].

According to the data of Li et al. [89], melatonin inhibited cell viability, suppressed colony formation, reduced cell migration and invasion, and induced cell apoptosis in pancreatic carcinoma cells. In addition, recent studies have shown that high doses (mM) of melatonin decreased pancreatic tumor cells proliferation and invasion by inhibition of NF-κB signaling pathway, but also enhanced gemcitabine cytotoxicity in these cells [100]. A supporting action of melatonin in antitumor therapy has also been demonstrated by Uguz et al. [101], who observed that melatonin enhances the antiproliferative effects of cytostatics such as 5-fluorouracil or cisplatin on pancreatic rat carcinoma. Co-treatment of cancer cells with these therapeutic agents in the presence of melatonin increased the population of apoptotic cells, and this effect was related to the increased production of reactive oxygen species [101]. Our recent unpublished results have also shown that combined treatment of PANC-1 cells with gemcitabine together with melatonin or its metabolite AFMK increased the therapeutic effects of gemcitabine. The results of our experiments together with the above-cited publications could suggest the possible future management of patients with pancreatic cancer with gemcitabine plus melatonin or its metabolite. Based on in vitro results, this combination could possibly give better perspectives for these patients, but future studies are required.

## 6. Conclusions

**Figure 1:**
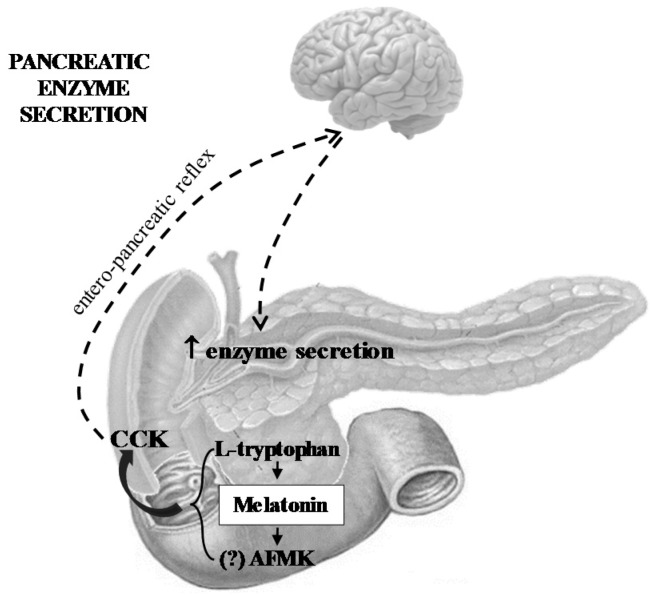
The effect of melatonin, its precursor l-tryptophan, and melatonin metabolite AFMK on pancreatic enzyme secretion. AFMK: *N*^1^-acetyl-*N*^1^-formyl-5-metoxykynuramine, CCK: cholecystokinin, dotted line arrow: nervous signal transmission via vago-vagal, entero-pancreatic reflex, up arrow: nervous signal delivery, bold arrow: CCK release stimulated by L-tryptophan, melatonin, and probably (?) by AFMK.

**Figure 2:**
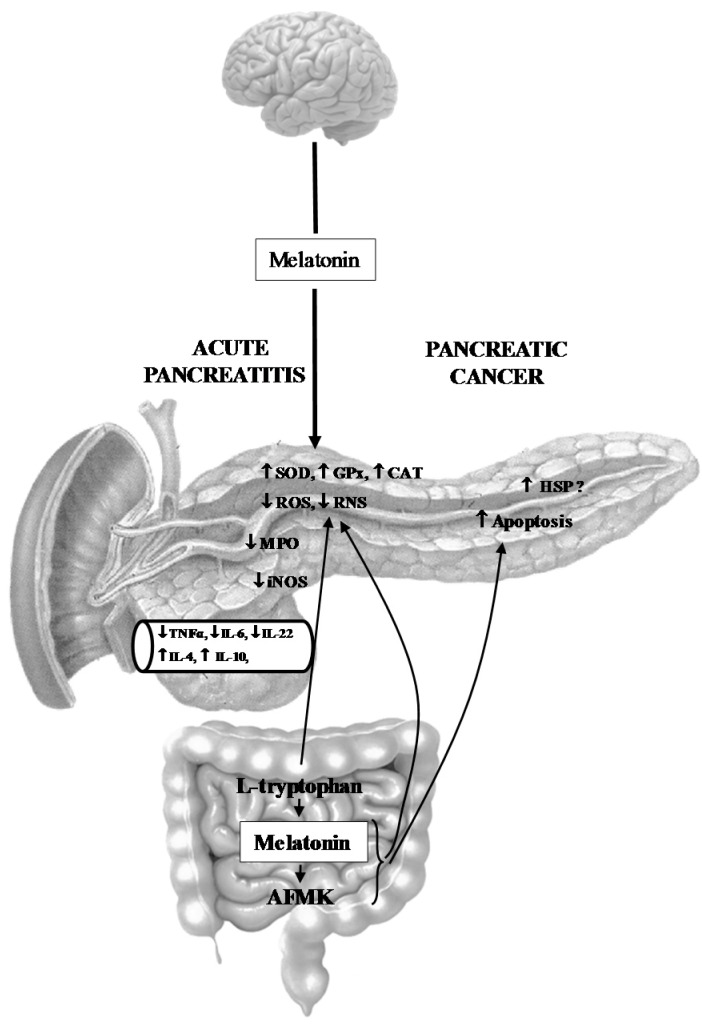
Effect of melatonin and its analogues on acute pancreatitis and on pancreatic tumorigenesis. CAT: catalase; GPx: glutathione peroxidase; HSP: heat shock protein; IL: interleukin; iNOS: inducible nitric oxide synthase; MPO: myeloperoxidase; ROS and RNS: reactive oxygen and nitrogen species; SOD: superoxide dismutase; TNFα: tumor necrosis factor α, AFMK: *N*^1^-acetyl-*N*^1^-formyl-5-metoxykynuramine, up arrow: effect of melatonin and its analogues on inflammatory mediators in acute pancreatitis and on molecular mechanism of pancreatic tumorigenesis, ?: possible activation of HSP by melatonin and AFMK, down arrow: pineal melatonin alleviated acute pancreatitis.

The melatonin system, including melatonin precursor l-tryptophan and the melatonin derivatives kynuramines, is probably implicated in pancreatic physiology and its malfunctioning, resulting in the impairment of pancreatic functions and pancreatic resistance to inflammation and cancer. The role of the melatonin system in the pancreas can be summarized as follows:

1. Melatonin and its related molecules may be involved in the physiological stimulation of pancreatic exocrine secretion dependent on the stimulation of CCK release and activation of duodeno-pancreatic neural reflex (Figure 1).
2. Melatonin might be implicated in the activation of the innate defense system of pancreatic protection, and its derivatives are responsible for a considerable part of the protective action of melatonin on the pancreas as part of its scavenging cascade (Figure 2).
3. Melatonin and its metabolites take part in the control of the increased production of heat shock proteins as well as in the signal transmission pathways modulating the process of apoptosis in pancreatic cancer cells, however its effect is not completely clear and requires further study (Figure 2).
